# Study on the Visualization of Pigment in *Haematococcus pluvialis* by Raman Spectroscopy Technique

**DOI:** 10.1038/s41598-019-47208-2

**Published:** 2019-08-20

**Authors:** Yongni Shao, Weimin Gu, Linjun Jiang, Yiming Zhu

**Affiliations:** 1 Shanghai Key Lab of Modern Optical System, University of Shanghai for Science and Technology No. 516, Jungong Road, 200093 Shanghai, China; 2 Shanghai Cooperation Innovation Centre of Terahertz Spectroscopy and Imaging Technology No. 516, Jungong Road, 200093 Shanghai, China; 3 College of Biosystems Engineering and Food Science, Zhejiang University, Hangzhou, 310058 China

**Keywords:** Biochemistry, Cellular imaging

## Abstract

As an ideal raw material for the production of astaxanthin, *H*. *pluvialis* was drawing attention during the last few years, there are some research topics initiated to find out the synthetic pathway of astaxanthin in *H*. *pluvialis*. In this study, confocal microscopic Raman technology was utilized to analyze the point-by-point mapping for *H*. *pluvialis*, and the visualization of pigment such as carotenoid and astaxanthin content were achieved. By comparing the Raman spectra of *H*. *pluvialis* and standard product of astaxanthin, and using the C = C stretching vibration of the Raman intensity as the main indicator for carotenoids, the visual spatial distribution for the carotenoids content was obtained. The MCR-ALS was applied to analyze the Raman data of *H*. *pluvialis*, and the information of astaxanthin was extracted to achieve real-time spatial distribution. The visualization of astaxanthin content shows that MCR-ALS is very effective for extracting the information of astaxanthin content from *H*. *pluvialis*. By exploring the spatial distribution of carotenoids and astaxanthin contents, analyzing the spatial distribution rules during its growth, Raman spectroscopy technology can be utilized to investigate the internal components of the pigment (ataxanthin, etc.) in *H*. *pluvialis*, which make it as an effective methodology to monitor the accumulation and changing mechanism of pigment content in microalgae.

## Introduction

Due to the high content of astaxanthin in *H*. *pluvialis*, the extraction of astaxanthin from *H*. *pluvialis* has become a hot topic worldwide. When *H*. *pluvialis* is under stress condition, the DNA, protein and cell membrane will damage due to the produced reactive oxygen species, and it will trigger the production of secondary carotenoids in order to reduce reactive oxygen^1^. In addition, it is reported that β-carotene is transported by chloroplast membrane and transformed into astaxanthin, and accumulated in lipid vesicles outside the chloroplast^2,3^.

Raman imaging is a powerful means of characterizing chemical information. The technology enables point-by-point scan different positions of a sample. By acquiring corresponding Raman spectra, pseudo-color images based on Raman information can be generated to show the structural information and distribution of components^4^. Raman spectroscopy and microscopic spectroscopy have recently been extended to microbiological studies. The combination of these two technologies makes it possible to study microbiological studies using microscopic Raman spectroscopy technology.

Each biomolecule in the microalgae has its own specific functional group, and their characteristic peak frequencies can be reflected in the Raman spectrum^5^. The characteristics of the functional groups make it feasible to obtain information on the chemical composition of organisms and analyze them *in situ*, which involves the study of pigment distribution and molecular structure of pigments in algal cells^6,7^, as well as the quantitative determination of lipids^8^. The visualization of the spatial distribution of components using specific functional groups corresponding to Raman information is a very effective way. When functional groups contained in multiple components are the same, chemometric is used to extract specific components information. Raman information about different components in algal cells can be discerned by MCR, which can help to achieve spatial distribution rules for algal cells^9^. MCR-ALS can decompose the mixture system without knowing its details in advance, it is able to distinguish and determine the corresponding curves (such as spectral curves, pH curves, system curves, etc.) or relative concentration distribution corresponding to the principal component, and it has been proved as an effective chemometric method^10,11^. MCR-ALS has been widely used in the analytic application of hyperspectral imaging^12^, chromatographic peaks, infrared spectroscopy, degradation processes in chemical reactions and so on^13^. Compared with the Principal Component Analysis (PCA), Independent Component Analysis (ICA) and other models, the analytical model of MCR-ALS can easily uncover the physicochemical information in the samples. By setting a few constraints for the obtained signal, such as bilinear, non-negative, unimodal, and closed, it is much easier to constrain and build the model based on known information so as to offer a more accurate solution.

Although some progress has been made in the visualization of carotenoids, research which can directly prove the transportation process in living cells is still missing. Mechanism of synthesis and accumulation of molecular and cellular of secondary carotenoids remained mysterious^14^. Reveal the accumulation mechanism of secondary carotenoids is of great significance for the research and could be very beneficial in the context of utilization of H. pluvialis. Therefore, it has become very meaningful to study the spatial distribution and change of components in H. pluvialis. In this work, the confocal micro-Raman technique was used to perform a point-by-point scan of H. pluvialis cells to obtain the Raman spectra of H. pluvialis, and the Raman spectra of H. pluvialis was analyzed. Firstly, the distribution map of carotenoids in algae cells was established by C = C stretching vibration Raman information. Then the Raman data was combined with MCR-ALS algorithm to distinguish the astaxanthin information. At the same time, the true content of astaxanthin was measured by HPLC. Finally, the relationship of the spatial distribution between carotenoids and astaxanthin in living cells was analyzed. This will serve as a reference for further analysis of the internal components of algae.

## Materials and Methods

### Culturing of H. pluvialis and stress conditions

Experimental H. pluvialis (strain 712) was purchased from the Wildlife Genebank of Chinese Academy of Sciences-Freshwater Algae. H. pluvialis was cultured in BG11 medium after autoclaving at 120 °C. The medium was placed in an artificial climate chamber. The incubator was set at a temperature of 25 °C. The light intensity was about 2500 Lux, and under periodic illumination conditions (12 light:12 dark) cultured continuously. The H. pluvialis was shaken three times a day, and the algae cells sinking at the bottom of the bottle were suspended in the culture solution. After the *H*. *pluvialis* was cultured for a period of time, *H*. *pluvialis* in a green cell state was centrifuged. Using a 50 mL centrifuge tube, the algal fluid was centrifuged at 5000 r/min at low temperature (4 °C) for 10 min. The supernatant was discarded and the algal cell pellet was washed twice with ultrapure water to remove the solute from the algal fluid. The algal cells were cultured in a BG11-N culture fluid and then put aside for the next step. Taking a 1000 mL Erlenmeyer flask after high-temperature sterilization, and choose the optimal combination of orthogonal experiment, namely sodium nitrate: 0.15 g/L, sodium acetate: 1.5 g/L, sodium chloride: 0.1%, finally prepare 1000 mL of algal fluid, the pH of the algae fluid is about 7.0, the algae density is about 2.65 × 10^5^ cells/mL. *H*. *pluvialis* was placed in a light incubator. Culture temperature was set at 25 °C, light intensity was set at 8000 Lux, and under periodic illumination conditions (24 light:0 dark).

### Confocal micro-raman spectroscopy data acquisition

In order to collect Raman spectra of living microalgae cells, cells were fixed in 2% to 4% agar. The agar powder was added to distilled water and heated until the solution boiling for 5 min, later it was left to cool down to about 40 °C, the algae liquid and the agar liquid were mixed in a volume ratio of 1:3. The cooled solidified mixture was sliced and the sample was placed on a slide. The microscope is used to identify single cells, an automatic XYZ platform was used to control the moving distance, so that Raman information can be collected for the entire single cell, achieving the entire surface scan on the cell. The prepared sample was fixed on a stage under the microscope objective of Renishaw in-Via-Reflex (London, England), a laser beam was emitted using a 532 nm argon ion (Ar+) laser device and focused on the surface of the sample through a 50× objective lens, and the incident laser power was approximately 1 mW. The exposure time was 1 s and the spectral collection range was 600–1800 Raman shift/cm^−1^. 3–4 sets of mapping data were acquired for different periods of the cells, acquisition step was 0.7 um, and the cumulative number of times was one.

### Experimental reagents and instruments

1. Experimental materials: agar, sodium nitrate, sodium acetate, sodium chloride, sodium hydroxide and dichloromethane were purchased from Sinopharm Chemical Reagent Co., Ltd., the purity was analytically pure; methanol was a chromatographically pure reagent (≥99.9%, Sigma); Astaxanthin Standard (CAS: 472-61-7, Aladdin); Ultrapure water.
2. Experimental equipment: Renishaw in-Via-Reflex (London, England), RXZ intelligent artificial climate chamber (Ningbo Jiangnan Instrument Factory, China), Heal Force Neofuge 15R desktop high-speed refrigerated centrifuge (Likang Biomedical Technology Holdings Co., Ltd., China), DKZ series electric thermostatic oscillation sink (Shanghai Yiheng Technology Co., Ltd., China), SCILDGEX centrifuge, Eppendorf 5418 high speed desktop centrifuge (Eppendorf, Germany), high performance liquid chromatography (Shimadzu, Japan), LDZX-50KBS vertical pressure steam sterilizer (Shanghai Shenan Medical Instrument Factory, China), single-channel pipette Eppendorf Research plus (Eppendorf, Germany), blood cell counting board.

### Determination of cell density and dry weight

Due to large volume of *H*. *pluvialis* cells, the cell density was usually determined by the blood count plate method. Cells were observed and counted on the counting plate with a ×40 objects glass. Before counting, the algae solution was shaken well, and the average was counted three times each time to reduce the counting error.

45 mL of algal fluid was sampled and centrifuged with a 50 mL centrifuge tube, and the cells were washed twice with distilled water to obtain an algal cell pellet. Then the precipitate was dried in an oven at 80 °C until the weight was stable and weighing. Weight of the algae cells was equal to the weighing result minus the weight of the test tube.

### HPLC determination of astaxanthin

1. Standard curve of astaxanthin: Dissolve 10 mg of astaxanthin in small portions methylene chloride to make 100 mg/L with methanol and stored in a brown bottle at −20 °C. The external standard method was employed for linear analysis. The appropriate volume of solution was extracted from the 100 mg/L standard solution to prepare 6 concentration gradients of 0.1 mg/L, 0.5 mg/L, 1 mg/L, 2 mg/L, 5 mg/L and 10 mg/L, respectively. Astaxanthin concent (mg/L) was set as the abscissa and the corresponding peak area was set as the ordinate. After the linear analysis, a standard curve of HPLC for astaxanthin was obtained in Fig. 1.

**Figure 1:**
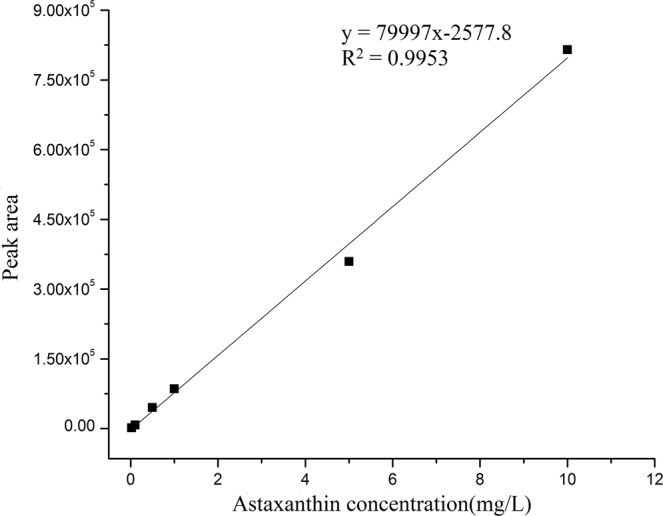
Standard curve of astaxanthin content measured by HPLC.
2. Extraction of astaxanthin: Take 3 mL of *H*. *pluvialis* algal fluid into a 5 mL test tube after spectral collection, then centrifuge for 12 min at 5000 r/min(4 °C), after centrifugation the supernatant was discarded. The algal cell sediment was washed with ultrapure water twice, appropriate amount of liquid nitrogen was added to the centrifuged test tube to make the algal cells hardened and easy to break the cell wall. After the liquid nitrogen was completely volatilized, 4 beads with a diameter of 5 mm were placed in the test tube and the cell wall was broken using a grinder. Then, adding 2 mL of a methanol-dichloromethane (Volume ratio 3:1) extract to the test tube. Oscillating for 10 s to fully extract astaxanthin, the extract was poured into a 10 mL test tube, and the extract was added to the grinding tube several times to reduce the residue of astaxanthin during grinding. The extracts were poured into 10 mL test tubes. The shaken extract was centrifuged at 10000 r/min for 15 min, and the supernatant was sealed and stored at −20 °C.
3. Astaxanthin ester saponification: Take 1 mL of freshly NaOH methanol solution(0.1 mol/L),Then add it to 5 mL of the extract and seal it in a dark refrigerator at 4 °C. After about 12 h of reaction, the sample was centrifugated for 15 min(10000 r/min, 4 °C). The supernatant was aspirated as a sample for HPLC analysis.
4. Chromatographic conditions: 250 × 4.6 mm chromatographic column (Agilent, America) was used and the mobile phase was methanol and water (95%:5%, v/v), the flow rate was 1.0 mL/min. Diode array detector was used to scan the sample and scanned-wavelength was 250–700 nm, 476 nm was set as the detection band. The sample size was 10 μL.

From the astaxanthin chromatogram, it was found to be about 8.337 min, and there is a distinct peak associated with astaxanthin. The chromatogram of Haematococcus pluvialis has a peak at about 8.855 min, which is very close to the peak of the standard. It can be considered that the peak at this position is the astaxanthin peak. The content of astaxanthin was determined from the size of the peak area.

### Raman spectroscopy preprocessing and modeling

When multiple spectra were mixed together, it would be difficult to identify cosmic rays that were not significant. Therefore, the Raman spectral set was preprocessed using De-trending, and many scanning points of the cosmic rays which are present in the Raman spectrum were revealed, then Wire Software was used to process the cosmic rays. Since the acquired spectrum has a good signal-to-noise ratio, the Nearest Neighbor method was used. After processing the spectrum containing cosmic rays, the background of the Raman spectrum needs to be removed. Baseline correction of Raman spectra was utilized, and then the Raman spectra were denoised and smoothed by Savitzky-Golay (SG)^15^. The subsequent model establishment was based on the data obtained after the above processing steps.

MCR was used to extract astaxanthin information from the Raman spectroscopy data^9^. The following is the main process of MCR-ALS algorithm operation^13^:

For a two-dimensional spectral matrix X (m × n), *m* is the number of samples and *n* is the number of wavelengths. X can be expressed as:

1 $${\rm{X}}={{\rm{CS}}}^{{\rm{T}}}+{\rm{E}}$$

In the formula: C(m × p) and S^T^ (p × n) represent the concentration distribution matrix and the pure spectrum combination matrix respectively, E represents the measurement error matrix, and p is the number of analysis system components. In order to distinguish the physical meanings of C and S, the multivariate curve resolution algorithm uses an alternating least-squares algorithm ALS to iteratively compute the approximate concentration distribution matrix and the initial value of the spectral matrix, and converts it into a true concentration distribution curve and spectral curve.

2 $${\rm{S}}={{\rm{X}}}^{{\rm{T}}}{{\rm{C}}({\rm{C}}}^{{\rm{T}}}{{\rm{C}})}^{-1}$$

3 $${\rm{C}}={\rm{XS}}{({{\rm{S}}}^{{\rm{T}}}{\rm{S}})}^{-1}$$

Normalize the *S* obtained by Eq. (2), that is, S = S/||S||, and then carry the normalized S into Eq. (3) and recalculate the estimate of C, Use the above two formulas to iteratively calculate S and C, until the degree of change between C and S obtained by two adjacent calculations converges, and C and S with physical and chemical significance were obtained. The initial value of C was obtained by the Evolving Factor Analysis (EPA). In the process of ALS iterative optimization, the spectrum was non-negatively restricted, and the concentration was non-negative and unimodal.

## Results

### Physiological changes of H. pluvialis

**Figure 2:**
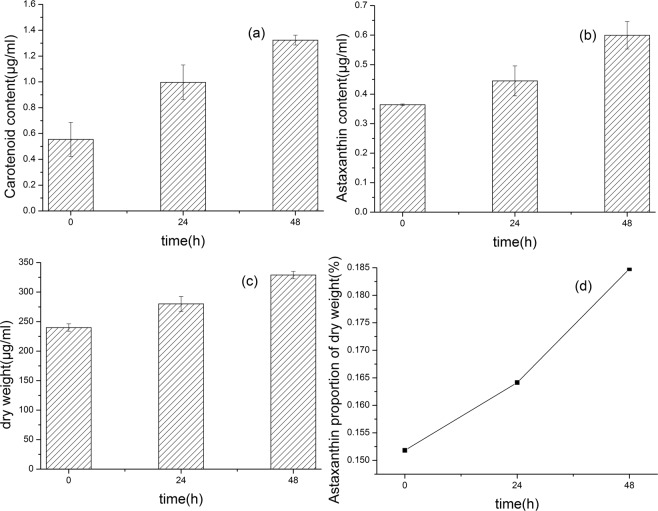
0–48 h Changes of related physiological indices of H. pluvialis. (**a**) Change in carotenoid content. (**b**) Changes in astaxanthin content. (**c**) Change in dry weight. (**d**) Change in astaxanthin proportion of single cell dry weight.

Figure 2 shows the change of carotenoid content, astaxanthin content and astaxanthin proportion of single cell dry weight in 0–48 h. In Fig. 2(a), carotenoid content was raised due to environmental stress. At the same time, astaxanthin content also increases as shown in Fig. 2(b). The increase in astaxanthin content of *H*. *pluvialis* also increased the dry weight of algae to some extent, as shown in Fig. 2(b,c). In Fig. 2(d), the average proportion of astaxanthin in the dry weight of unicellular gradually increased, ranging from 0.152%(0 h) to 0.185%(48 h), and the proportion of 0 h was similar to the ratio of 0.164% at 24 h, indicating that From 0–24 h, accumulation of astaxanthin in the *H*. *pluvialis* cells was slow, probably due to the self-regulatory response time required for changes in the external environment within the algal cells.

### Raman spectroscopy treatment of *H*. *pluvialis* cell

The Raman spectra of 1444 scanning spots were obtained by confocal microscopic Raman spectrometer from *H*. *pluvialis* cell samples. Raman spectrum acquired from scanning points in different positions has certain differences. The spectrum with weak Raman intensity may represent the content of the scan sites with low content or only background information. At some Raman shifts, such as 980–1050 cm^−1^, 1100–1230 cm^−1^, and 1450–1560 cm^−1^, there are more pronounced intensity peaks.

For the original Raman spectral curve collected, first use the detrending algorithm to preprocess, then remove the cosmic ray and correct baseline, finally use the convolution smoothing method to smooth the Raman spectrum to denoise. The following model establishment data were based on the above processing steps.

**Figure 3:**
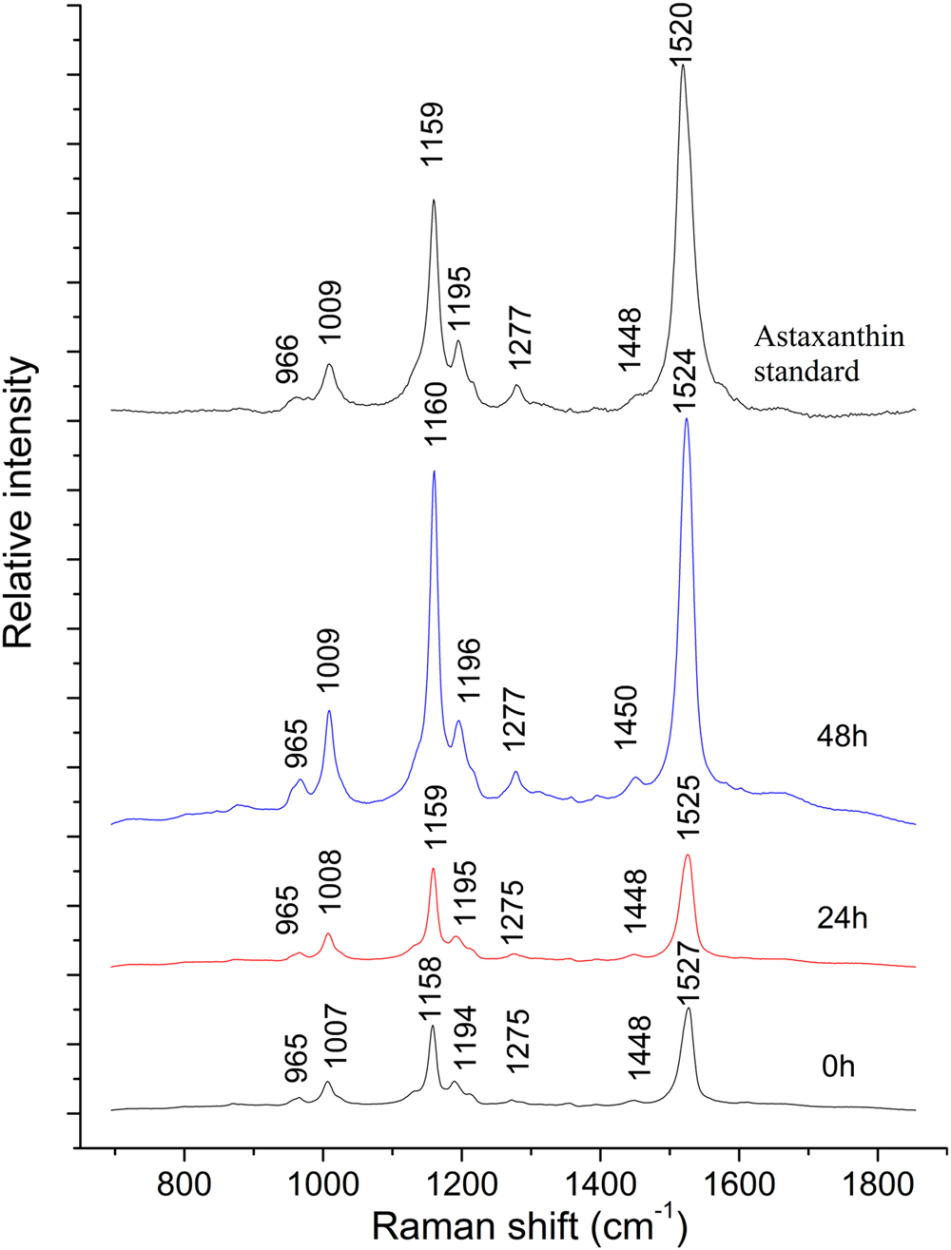
Raman spectra of astaxanthin standard and *H*. *pluvialis* (0–48 h).

Figure 3 shows the Raman average spectra of *H*. *pluvialis* from 0–48 h and the standard curve of astaxanthin. For the Raman curve of the astaxanthin standard, it can be found that there are many Raman peaks in the Raman spectrum of the standard, with strong Raman peaks at Raman shifts of 1009 cm^−1^ (C-H bend), 1159 cm^−1^ (C-H stretch) and 1519 cm^−1^ (C = C stretching vibration)^16^. There are other weaker peaks, of which 1448 cm^−1^ (C-H_2_), 1195 cm^−1^ (C-H_3_), 1278 cm^−1^ (C-H_3_ and C-C) and 966 cm^−1^ (C-H_3_)^17–21^. Using the information of these bonds, the correlation between Raman spectra of *H*. *pluvialis* and astaxanthin can be analyzed. The visualization map of *H*. *pluvialis* cells at 0–48 h were obtained by removing the irrelevant points in the Raman scan. The Table 1 shows the corresponding substance of the related bonds in Raman spectra for *H*. *pluvialis*. It can be seen from the Fig. 3 that the Raman spectrum of *H*. *pluvialis* is very similar to the Raman spectrum of the astaxanthin standard measured, and the three average spectra all have obvious Raman peaks. Among them, the three peaks at 1007–1009 cm^−1^, 1158–1160 cm^−1^, and 1524–1527 cm^−1^ are the most noticeable; the two strong peaks at 1007–1009 cm^−1^, 1158–1160 cm^−1^ and other relatively weak peaks have only 1–2 cm^−1^ deviation from the peaks of the Raman peak corresponding to astaxanthin. By the displacement of these similar peaks, it can be inferred that some changes have occurred in the internal components of *H*. *pluvialis*. It can be seen that from 0–48 h, the peak intensity of three corresponding Raman lines at 1007–1009 cm^−1^, 1158–1160 cm^−1^ and 1524–1527 cm^−1^ increases with time. Through the analysis of key position information, this change may be mainly caused by the increase of carotenoids, while carotenoid mainly contains other pigments such as β-carotene, astaxanthin, etc. According to the synthetic pathway of astaxanthin, the accumulation of astaxanthin requires the synthesis of carotenoids such as β-carotene, so the main reason that may cause this change is the increase in astaxanthin. However, the Raman intensity at 1007–1009 cm^−1^, 1158–1160 cm^−1^ and 1194–1196 cm^−1^ may also be affected by other components (lipids, proteins, amide III, etc.). Therefore, it is more meaningful to select 1524–1527 cm^−1^ (C = C) to analyze the change of carotenoids.

**Table 1:** The peaks of *H*. *pluvialis* with corresponding bonds and substances.

Raman shift(cm^−1^)	Bonds	Substances	References
965–966	C-H_3_	/	^17^
1007–1009	C-H bend, C-H_3_,C-C	Related carotenoids, β-carotene, astaxanthin, lipids, etc.	^16, 18^
1157–1160	C-C stretching vibration	Related carotenoids, β-carotene and proteins	^16, 18^
1194–1196	C-H deformation vibration	β-carotene, amide III	^19^
1275–1277	C-C	Amide III	^20^
1448–1450	C-H_2_ deformation vibration	Unsaturated fatty acid	^6, 21^
1516–1527	C = C stretching vibration	Astaxanthin, β-carotene, related carotenoids	^21, 22^

### Analysis on visualization of carotenoids and astaxanthin

In the average spectra of the three stress stages(0 h, 24 h, 48 h) of *H*. *pluvialis* in Fig. 3, their corresponding C = C stretching vibration at the Raman peak of 1524–1527 cm^−1^ is the main sensitive marker of the chemical structure of carotenoids^7^. Therefore, Raman information in 1524–1527 cm^−1^ can be used to analyze changes in astaxanthin. It can be seen that the Raman shifts(C = C) of *H*. *pluvialis* at 0–48 h are all different, and there is a deviation of 4–7 cm^−1^ with 1520 cm^−1^ (C = C) for the astaxanthin standard, the Raman shift (C = C) start blue-shifted with the accumulation of astaxanthin. The Raman spectrum of *H*. *pluvialis* is a biological spectrum. In addition to astaxanthin, other carotenoids (such as β-carotene) in the *H*. *pluvialis* cells also have C = C stretching vibration^21,22^, multiple components information superposition also caused the key position offset. Therefore, the C = C stretching vibration strength obtained in Raman spectrum of *H*. *pluvialis* cells mainly reflects the information of the carotenoids composition. The Raman information of the C = C stretching vibration can be used as a marker of the Raman mapping of the carotenoids mixture in this study. The mapping intensity can be visualized using the peak intensity at the position of the displacement of the tensile bond to achieve the carotenoids distribution analysis. As a substance in carotenoids, astaxanthin needs further analysis of the acquired Raman spectrum in order to extract the effective information.

**Figure 4:**
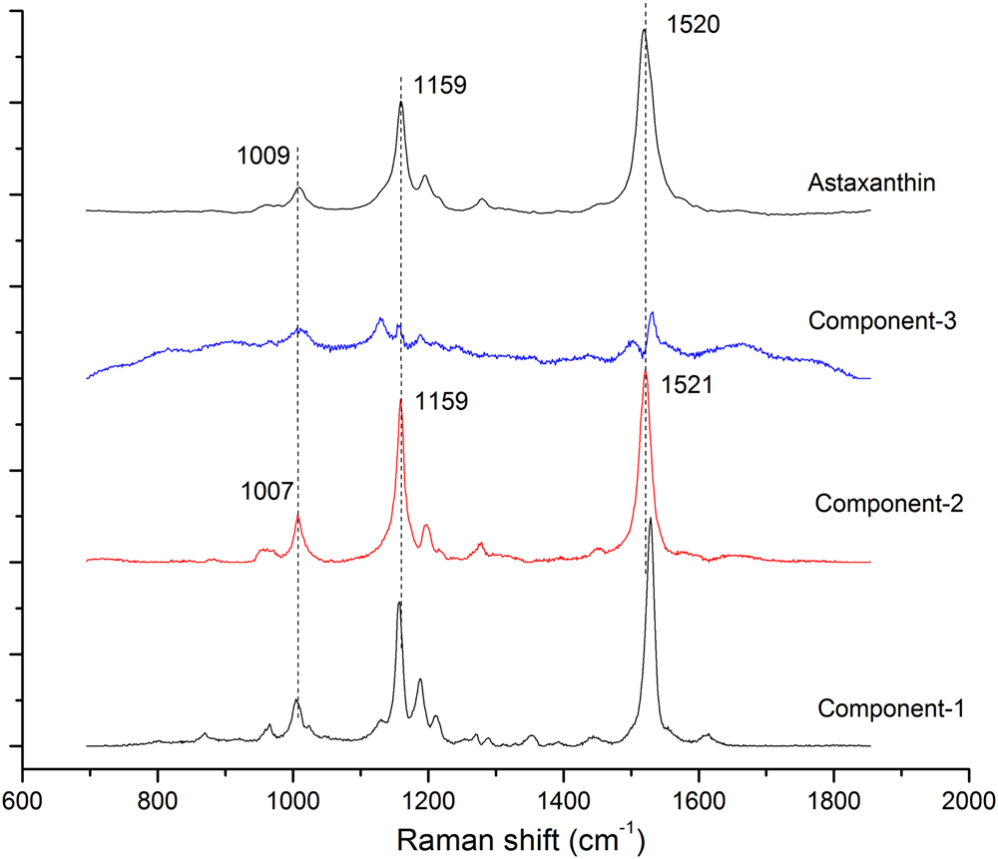
Raman spectroscopy of *H*. *pluvialis* based on MCR-ALS (three main Components) and astaxanthin standard.

It is difficult to distinguish the astaxanthin information. The components were resolved after 50 iterations by MCR-ALS from the Raman spectrum of *H*. *pluvialis* and the spectra of the three principal components(Component-1, Component-2, Component-3) were compared with the Raman spectra of astaxanthin standards. As shown in Fig. 4, it can be found that the spectral characteristics of Component-2 was very similar to the astaxanthin standard., the peak position of the three major peaks are very close and the corresponding peak position at the C = C stretching vibration was only about 1 cm^−1^ away from the astaxanthin standard. By comparison and analysis, Component-2 can be presumed to be astaxanthin in *H*. *pluvialis*. After deducing that Component-2 was astaxanthin, the concentration of the components can be calculated in combination with MCR-ALS.

Calculate the ratio of Component-2 to total concentration and the ratio was combined with the Raman peak intensity of the pretreated *H*. *pluvialis* Raman spectrum at the corresponding C = C stretching vibration(1524–1527 cm^−1^), then the intensity value of astaxanthin in *H*. *pluvialis* was calculated and the obtained intensity value was used to perform visual analysis of astaxanthin in cells. The same data processing method was used to process data of 24 h and 48 h to obtain the Raman spectrum information of astaxanthin.

**Figure 5:**
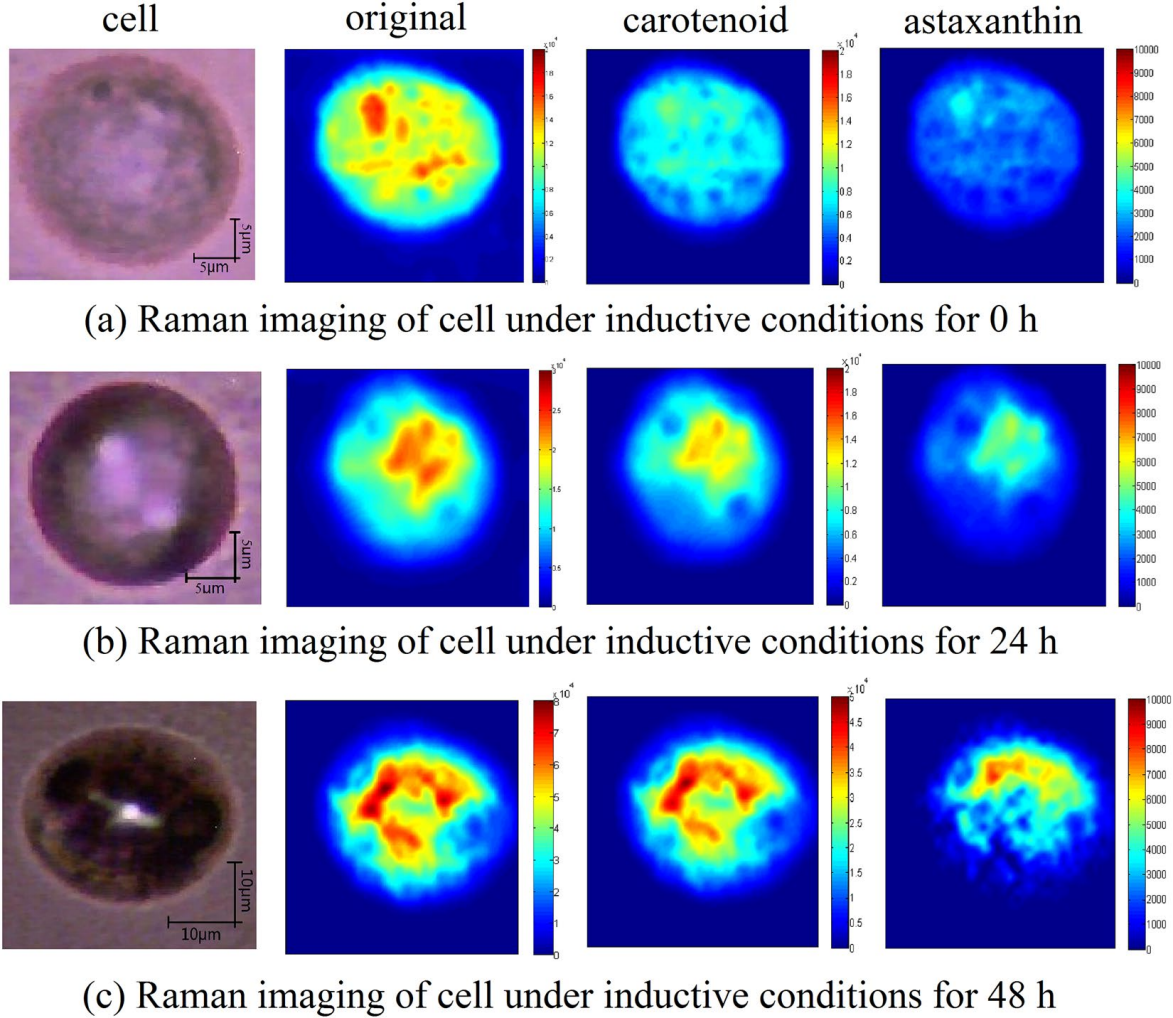
Concentration maps of spectral components representing carotenoids and astaxanthin in H. pluvialis cells (Note: Distribution of carotenoid was obtained by raman intensity at the C = C stretch bond, and distribution of astaxanthin was obtained by raman intensity at the C = C stretch bond combined with MCR-ALS model). (**a**–**c**) Raman images of cells under inductive conditions for 0, 24, 48, respectively.

After scanning the cell of *H*. *pluvialis* by confocal Raman microscopy, a visual image of carotenoids was established with the Raman peak intensity of the C = C stretching vibration. Combined with the MCR-ALS to analysis astaxanthin, the corresponding concentration of astaxanthin was introduced to achieve spatial visualization in cells. As shown in Fig. 5, the spatial distribution of carotenoids and astaxanthin was visualized. From Fig. 5, it can be seen that the inverted carotenoid content map has basically formed a cell shape, indicating that in the *H*. *pluvialis* cells, the carotenoids are widely distributed, but the location of the distribution is also different; During the stress period from 0–48 h, astaxanthin is rapidly produced and accumulated around the nucleus during intermediate stages to protect the ultrastructures^23^. It is esterified in the endoplasmic reticulum and finally deposited in the cytoplasmic lipid droplets, but not in the thylakoid membrane^24^. At the same time, it can be seen from the Fig. 2(a,b) that the content of carotenoid and astaxanthin gradually change from 0–48 h, the intensities were found to be initially concentrated towards the cell center and then extended to the cell periphery^25^. As shown from Fig. 2(d), the astaxanthin proportion of dry weight in single cell has been increasing, indicating that the astaxanthin visualization map can reflect the change of astaxanthin in the whole cell to some extent. Therefore, the increase in intensities and the expansion of the distribution in visualization map indicate that the content of astaxanthin was rising. Comparing the visual distribution maps between astaxanthin and carotenoids, it can be found that astaxanthin is basically distributed in the space where carotenoids are distributed, and in the carotenoid map, where raman intensity is higher, which is also reflected in the visual map of astaxanthin. Therefore, it can also be inferred from the two diagrams that in the carotenoid diagram, the higher content sites may be partially caused by astaxanthin, Thus, it can be analyzed that astaxanthin has a certain contribution rate to the Raman peak value of the C = C stretching vibration in Raman spectra of *H*. *pluvialis*. At the same time, it was demonstrated that the MCR-ALS method is very effective in extracting information of astaxanthin from *H*. *pluvialis*, and is of great significance for future research on other components of *H*. *pluvialis*. Visualization of carotenoids reflected the intracellular changes of *H*. *pluvialis* during the stress process, indicating that the Micro-Raman technique is effective for the detection of internal components of algal cells. It provides a very direct evidence for the study of the internal composition of the algal cells and is beneficial to explore the biological characteristics of living cells.

## Discussion

2–3 different cells were measured and analyzed each time, but considering the consistenttrend of results, we only present the images of one cell as a typical case. In order to verify the visualized results of the test, we measured the actual content of carotenoids and astaxanthin. Comparing the components resolved by MCR-ALS with the Raman spectra of astaxanthin standards, Component-2 was deemed to be the information of astaxanthin in the pigment mixture. The visual representation of Component-2 can be used as an efficient indicator to reflects the information on astaxanthin in the cells.

This article provides the visual analysis of carotenoids and astaxanthin. While *H*. *pluvialis* contains many other ingredients such as β-carotene, oil, etc. These ingredients also shows certain relationship with the change of astaxanthin content. The multi-component study should be explored and taken into the investigation in the future to provide more direct evidence for the astaxanthin synthesis pathway, so that the relationship between them and astaxanthin can be directly analyzed from these changes. It is also essential to improve and mature the level of technology and eventually achieve to a higher level of application such as dynamic monitoring of compositional changes in living cells. The current research is with quite a lot technical challenges. Although each shift has corresponding bond to analyze the component information, the Raman spectrum of the living body contains various components, so that how to distinguish the components from each other becomes problematic. At present, there are many approaches such as multivariate linear resolution which can analyze the spectral information of components. However, these methods are built on mathematical theory. Investment on more effective and accurate analytical methods is foreseen to attract more attention and will be explored in our future study.

## Conclusion

The imaging analysis of carotenoids was achieved by using the confocal micro Raman technique to analyze point-by-point imaging *H*. *pluvialis* cells. Through the comparison of the two distribution images, it was revealed that accumulation of astaxanthin had a certain effect on the increase of carotenoids. By comparing the difference between Raman spectra of *H*. *pluvialis* and astaxanthin standards, the Raman intensity at the C = C stretching vibration position was used as the main indicator of carotenoids to obtain the carotenoid distribution of *H*. *pluvialis*. The MCR-ALS are utilized for iterative modeling to distinguish the astaxanthin information in Raman spectra of *H*. *pluvialis* and this information then can be used to achieve visualization of the spatial distribution of astaxanthin in *H*. *pluvialis* cells. At the same time, the visual image of astaxanthin shows that MCR-ALS is very beneficial for the extraction of component information in the *H*. *pluvialis* astaxanthin. By exploring the spatial distribution of carotenoids and astaxanthin, and analyzing the relevant laws from the spatial distribution images, it is demonstrated that the Micro-Raman spectroscopy technique is feasible for studying the internal components of *H*. *pluvialis* cells. In the future research, it is necessary to explore the relationship between more components in order to expect more complete and rich visual evidence in the astaxanthin accumulation synthesis pathway.

As a non-destructive detection method, Micro-Raman spectroscopy technology has extremely vital meanings for the detection of living cells. Using this technology can not only achieve two-dimensional imaging analysis, but also can achieve three-dimensional analysis of cell components^26^, realizing multidimensional analysis of components in living cells. Of course, there are also some disadvantages. In this study, Raman information of the whole cell collected point by point takes a long time. In worst case, time-consuming is close to one hour, and the shortest one takes half an hour. Therefore, this deficiency limits the rapid detection of Raman spectroscopy to some extent. However, Raman technology has been maturing, and rapid imaging of Raman has become possible in recent years. Making use of coherent anti-Stokes Raman scattering and spontaneous Raman spectroscopy can achieve high-throughput cell detection^27^. Raman imaging technology can achieve a direct overall presentation of objects^28^, thereby increasing the speed of measurement. Due to the complexity of the components of the organism, the collected Raman spectrum may contain many non-target components. At present, the Raman standard atlas is very scarce, which also brings certain difficulties for the analysis of biological components. Although some methods for compositional analysis are proposed much more frequently, they are all based on mathematical theory, and the judgment of the pure material spectrum still requires some experience. However, as the chemometrics approach matures, studies of biological components will not stagnate^9^. Raman spectroscopy still has great potential for the study of organisms.

## References

[1] Li Y, Sommerfeld M, Chen F, Hu Q (2008). Consumption of oxygen by astaxanthin biosynthesis: a protective mechanism against oxidative stress in *H*. *pluvialis* (Chlorophyceae). J. Plant. Physiol..

[2] Fátima Santos M, Mesquita JF (1984). Ultrastructure study of Haematococcus lacustris (Girod) Rostafinski (Volvocales) I. Some aspects of carotenogenesis.Cytologia..

[3] Grünewald K, Hirschberg J, Hagen C (2001). Ketocarotenoid biosynthesis outside of plastids in the unicellular green alga *H*. *pluvialis*. J. Biol. Chem..

[4] Heraud P (2010). Effects of pre-processing of Raman spectra on *in vivo* classification of nutrient status of microalgal cells. J. Chemometr..

[5] Wei X (2014). Microalgal detection by Raman microspectroscopy. Trac-Trend. Anal. Chem..

[6] Kaczor A, Turnau K, Baranska M (2011). *In situ* Raman imaging of astaxanthin in a single microalgal cell. Analyst..

[7] Kaczor A, Baranska M (2011). Structural changes of carotenoid astaxanthin in a single algal cell monitored *in situ* by Raman spectroscopy. Anal. Chem..

[8] Ota S (2010). Raman microspectroscopy of individual algal cells: sensing unsaturation of storage lipids *in vivo*. Sensors..

[9] Collins AM (2011). Carotenoid Distribution in Living Cells of *H*. *pluvialis* (Chlorophyceae). Plos One..

[10] Tauler R, Kowalski B, Fleming S (1993). Multivariate curve resolution applied to spectral data from multiple runs of an industrial process. Anal. Chem..

[11] Tauler R, Lacorte S, Barceló DJ (1996). Application of multivariate self-modeling curve resolution to the quantitation of trace levels of organophosphorus pesticides in natural waters from interlaboratory studies. J. Chromatogr. A..

[12] Zhang X, Zhang ZY (2016). Multivariate Curve Resolution Alternating Least Squares with Shape Smoothness for the analysis of Hyperspectral Imaging. Spectrosc. Spect. Anal..

[13] Hou LL, Ren HX, Liu Y, Shen Q (2012). Study of characterization and degradation process of curcumin using multivariate curve resolution-alternating least squares. Comput. Appl. Chem..

[14] Abbas A, Josefson M, Abrahamsson K (2011). Characterization and mapping of carotenoids in the algae Dunaliella and Phaeodactylum using Raman and target orthogonal partial least squares. Chemometr. Intell. Lab..

[15] Zhang D, Benamotz D (2000). Enhanced chemical classification of raman images in the presence of strong fluorescence interference. Appl. Spectrosc..

[16] Sharma SK (2015). An integrative Raman microscopy-based workflow for rapid *in situ* analysis of microalgal lipid bodies. Biotechnol. Biofuels..

[17] Huang WE, Li M, Jarvis RM, Goodacre R, Banwart SA (2010). Shining light on the microbial world the application of Raman microspectroscopy. Adv. Appl. Microbiol..

[18] Wang X (2012). Analysis of astaxanthin in Phaffia rhodozyma using laser tweezers raman spectroscopy. Spectrosc. Spect. Anal..

[19] Maquelin K (2002). Identification of medically relevant microorganisms by vibrational spectroscopy. J. Microbiol. Meth..

[20] Schuster KC, Reese I, Urlaub E, Gapes JR, Lendl B (2000). Multidimensional Information on the Chemical Composition of Single Bacterial Cells by Confocal Raman. Microspectroscopy. Anal. Chem..

[21] Wood BR (2005). A portable Raman acoustic levitation spectroscopic system for the identification and environmental monitoring of algal cells. Anal. Chem..

[22] Liu J, Huang Q (2016). Screening of Astaxanthin-Hyperproducing *H*. *pluvialis* Using Fourier Transform Infrared (FT-IR) and Raman Microspectroscopy. Appl. Spectrosc..

[23] Saha Sushanta Kumar, McHugh Edward, Hayes Jeremiah, Moane Siobhan, Walsh Daniel, Murray Patrick (2013). Effect of various stress-regulatory factors on biomass and lipid production in microalga Haematococcus pluvialis. Bioresource Technology.

[24] Li, K. *et al*. *In vivo* kinetics of lipids and astaxanthin evolution in, Haematococcus pluvialis, mutant under 15% CO_2_, using Raman microspectroscopy. Bioresource. Technol. S0960852417306247 (2017) 10.1016/j.biortech.2017.04.116 28533068

[25] Solovchenko, Alexei E (2015). Recent breakthroughs in the biology of astaxanthin accumulation by microalgal cell. Photosynth. Res..

[26] Marina W (2013). Three-Dimensional Ultrastructural Study of Oil and Astaxanthin Accumulation during Encystment in the Green Alga *H*. *pluvialis*. Plos One..

[27] He XN (2012). Coherent anti-Stokes Raman scattering and spontaneous Raman spectroscopy and microscopy of microalgae with nitrogen depletion. Biomed. Opt. Express..

[28] Ling J, Weitman SD, Miller MA, Moore RV, Bovik AC (2002). Direct Raman imaging techniques for study of the subcellular distribution of a drug. Applied Optics..

