# Publisher Correction: Study on the Visualization of Pigment in *Haematococcus pluvialis* by Raman Spectroscopy Technique

**DOI:** 10.1038/s41598-020-58427-3

**Published:** 2020-02-05

**Authors:** Yongni Shao, Weimin Gu, Linjun Jiang, Yiming Zhu, Aiping Gong

**Affiliations:** 1 Shanghai Key Lab of Modern Optical System, University of Shanghai for Science and Technology No. 516, Jungong Road, 200093 Shanghai, China; 2 Shanghai Cooperation Innovation Centre of Terahertz Spectroscopy and Imaging Technology No. 516, Jungong Road, 200093 Shanghai, China; 3 College of Biosystems Engineering and Food Science, Zhejiang University, Hangzhou, 310058 China; 4 College of Intelligent Manufacturing and Equipment, Shenzhen Institute of Information Technology, Shenzhen, Guangdong 518172 China

Correction to: *Scientific Reports* 10.1038/s41598-019-47208-2, published online 20 August 2019

Aiping Gong was omitted from the author list in the original version of this Article. As a result, Yongni Shao was incorrectly listed as the corresponding author. The correct corresponding author for this Article is Aiping Gong. Correspondence and request for materials should be addressed to gongap@sziit.edu.cn. This has been corrected in the PDF and HTML versions of the Article.

The Author Contributions section now reads:

“The work presented here was carried out in collaboration between all authors. YS conceived the idea and drafted the manuscript. WG, LJ co-worked on associated data collection, data analysis and carried out the experimental work. YZ have supported the instrument and technology. AG subsequently revised the paper and sponsored the publication of the paper.”

